# Case Report: Responsive Neurostimulation of the Centromedian Thalamic Nucleus for the Detection and Treatment of Seizures in Pediatric Primary Generalized Epilepsy

**DOI:** 10.3389/fneur.2021.656585

**Published:** 2021-04-28

**Authors:** William P. Welch, Jasmine L. Hect, Taylor J. Abel

**Affiliations:** ^1^Division of Pediatric Neurology, Department of Pediatrics, University of Pittsburgh School of Medicine, Pittsburgh, PA, United States; ^2^Department of Neurological Surgery, University of Pittsburgh School of Medicine, Pittsburgh, PA, United States; ^3^Department of Bioengineering, University of Pittsburgh Swanson School of Engineering, Pittsburgh, PA, United States

**Keywords:** case report, responsive neurostimulation, drug-resistant epilepsy, centromedian nucleus, pediatric generalized epilepsy, absence seizures

## Abstract

Up to 20% of pediatric patients with primary generalized epilepsy (PGE) will not respond effectively to medication for seizure control. Responsive neurostimulation (RNS) is a promising therapy for pediatric patients with drug-resistant epilepsy and has been shown to be an effective therapy for reducing seizure frequency and severity in adult patients. RNS of the centromedian nucleus of the thalamus may help to prevent loss of awareness during seizure activity in PGE patients with absence seizures. Here we present a 16-year-old male, with drug-resistant PGE with absence seizures, characterized by 3 Hz spike-and-slow-wave discharges on EEG, who achieved a 75% reduction in seizure frequency following bilateral RNS of the centromedian nuclei. At 6-months post-implant, this patient reported complete resolution of the baseline daily absence seizure activity, and decrease from 3–4 generalized convulsive seizures per month to 1 per month. RNS recordings showed well-formed 3 Hz spike-wave discharges in bilateral CM nuclei, further supporting the notion that clinically relevant ictal discharges in PGE can be detected in CM. This report demonstrates that CM RNS can detect PGE-related seizures in the CM nucleus and deliver therapeutic stimulation.

## Introduction

Primary generalized epilepsy (PGE) accounts for ~15–20% of all children diagnosed with epilepsy (1, 2). Of patients with PGE, 10–20% will meet criteria for drug-resistant epilepsy (3). Unfortunately, there are no FDA-approved neuromodulation treatment options for PGE. Absence seizures are commonly seen in patients with PGE in the form of behavioral arrest with impaired awareness, with concomitant variable motor or behavioral manifestations. Uncontrolled seizures are a significant source of morbidity in PGE, impacting development, academic performance, activities of daily living, and quality of life measures (4–7). Investigation and validation of neuromodulation treatment options for pediatric PGE are necessary to improve patient outcomes and quality of life.

Stimulation of the thalamic centromedian nucleus (CM) is associated with improved frequency and severity of generalized seizures in adult patients, including both deep brain (DBS) stimulation and responsive neurostimulation (RNS) system approaches (8, 9). There is supportive evidence for the use of deep brain stimulation (DBS) in pediatric epilepsy, although data is limited (10, 11). A limitation of DBS devices is that while they are able to deliver programmed stimulation in an open-loop system, they lack the functionality to record or respond to changes in brain activity and, therefore, cannot be programmed to deliver personalized therapy in response to patient-specific seizure patterns (12). The closed-loop RNS system has the functionality of recording and storing patient-specific neuronal activity and can be programmed to deliver stimulation in response to detected changes during seizure activity. RNS has been shown to be safe and effective in the treatment of drug-resistant epilepsy (13, 14). Here, we report the diagnostic utility and outcome of bilateral CMN thalamic RNS implantation in a single pediatric patient for the treatment of PGE.

## Patient Information

A 16-year-old male diagnosed with childhood absence epilepsy (CAE) at 4 years of age presented for evaluation for uncontrolled seizures. At the time of diagnosis, he was an otherwise healthy and developmentally appropriate child with no family history of significant neurological disease or parental consanguinity. Genetic testing was not performed at our institution during his evaluation. Initial seizure semiology consisted of behavioral arrest, eye rolling, and variable impaired awareness, with rare progression to bilateral tonic-clonic seizure. Initial EEG captured typical absence seizures with correlating 3 Hz spike-and-slow-wave discharges, as well as interictal high-amplitude spike- and polyspike-and-slow-wave discharges. Repeat EEG over several years remained consistent with this diagnosis. Seizures proved resistant to treatment with ethosuximide, lamotrigine, topiramate, clobazam, valproate, and the modified Atkin's diet. At 12 years of age, repeat brain MRI detected a lesion in the right amygdala suggestive of dysembryoplastic neuroepithelial tumor (DNET). Repeat routine EEG while on medications again demonstrated generalized spike- and polyspike-and-slow-wave discharges and typical absence seizures, with new findings of independent bilateral centroparietal and centrotemporal epileptiform discharges. Additionally, a focal impaired awareness seizure with temporal semiology was captured on prolonged EEG which was electroclinically distinct from his typical absence seizures, with onset characterized by rhythmic theta activity over the left temporal head region and clinical accompaniment speech difficulty, confusion, and oral automatisms lasting over 9 min.

Due to new neuroradiologic and EEG findings, phase 2 pre-surgical evaluation was pursued. Fourteen sEEG electrodes were implanted targeting the right temporal lobe (including the right amygdala lesion) and cingulate, and left hippocampus. Prior to and during weaning of anti-seizure medications, numerous typical absence seizures were captured. Electrographic onset was not localizable, with diffuse onset of 2.5–3.0 Hz spike-wave morphology throughout the intracranial array, including the bilateral hippocampal electrodes. Interestingly, independent rare bursts of 2.5–3.0 Hz spike-wave discharges were detected in peri-lesional contacts in the right amygdala, but never evolving to electrographic seizures.

Robot-assisted stereotactic biopsy of the amygdala lesion was performed at the time of sEEG electrode removal, which was negative due to small sample size. However, given the progression of the lesion and presence of peri-lesional epileptiform activity, stereotactic laser ablation (SLA) of the lesion was performed with simultaneous redo stereotactic biopsy. The redo biopsy was consistent with a low grade glioneuronal neoplasm, however given the small volume of the biopsy, a more specified diagnosis (e.g., DNET was not achieved). A complete ablation of the lesion was achieved. After surgical recovery, the patient continued to have daily typical absence seizures, with occasional progression to bilateral tonic-clonic seizure, despite continuation of prior anti-seizure medications. Given continuance of seizures despite best medical management, vagus nerve stimulation (VNS) was discussed with the family, who were not interested in VNS. CM RNS was thus offered to the family and after discussion of the risks and potential benefits the patient and family elected to proceed.

## Therapeutic Intervention

**Figure 1:**
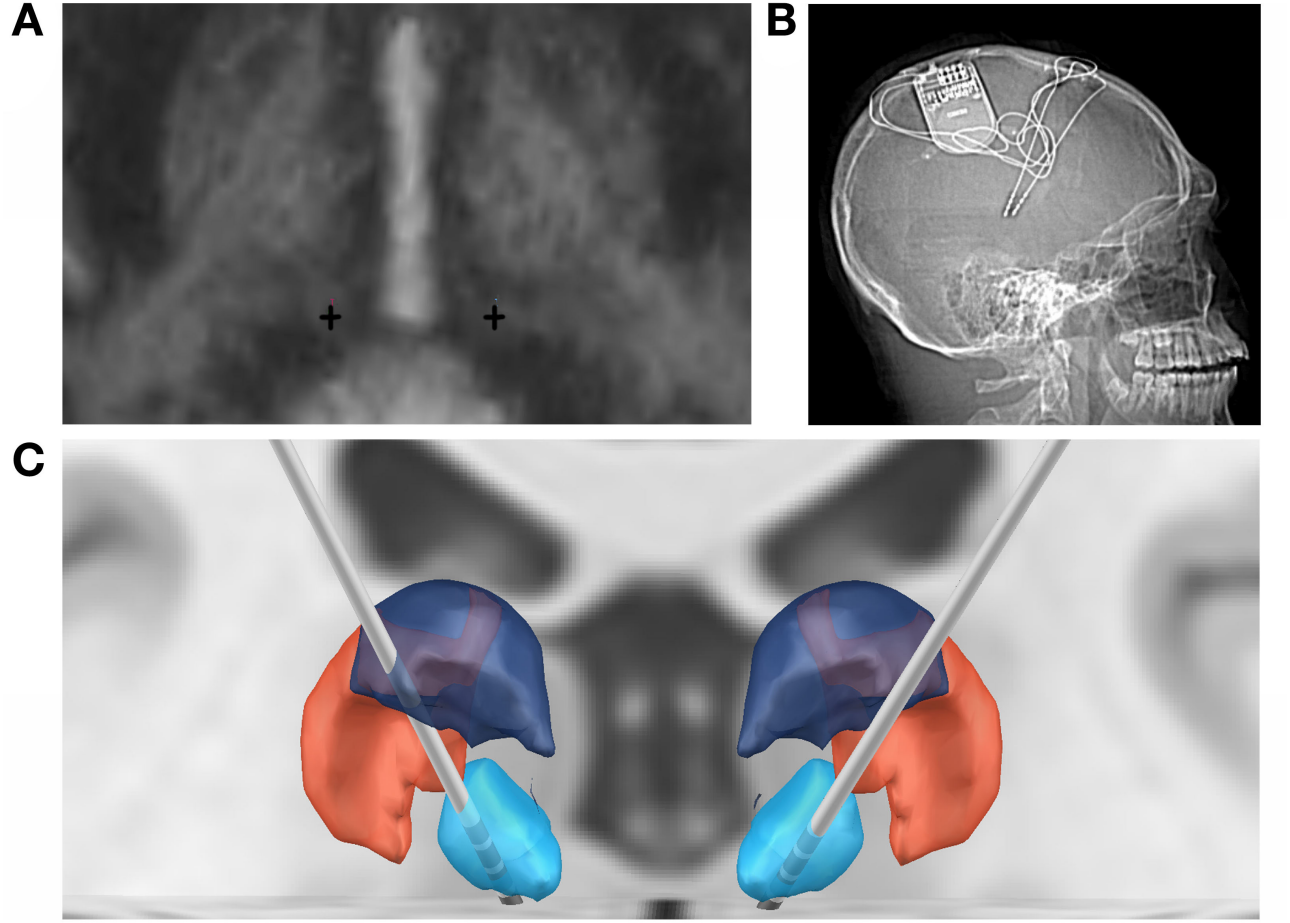
Bilateral RNS CMN implantation. **(A)** Direct targeting of the bilateral CMN (crosses) in AC-PC orientation. **(B)** Post-implant x-ray of RNS system. **(C)** Coronal 3D reconstruction of bilateral RNS implantation targeting the CMN (blue), in relation to the posterior part of the ventral posterolateral nucleus (VPLp; red) and posterior dorsal part of the ventral lateral nucleus (VLpd; purple).

The patient was taken to the operating room for implantation of bilateral CM RNS electrodes. CM targeting was performed using indirect and direct targeting as previously described (10, 15–17). Briefly, MP2RAGE inversion images were merged to a preoperative thin-cut (1 mm) CT angiogram using the ROSA platform. Standard entry points near the coronal suture that would allow an avascular trajectory to the target were selected. Four-contact depth electrodes, with 3.5-mm spacing, were implanted with these trajectories using the ROSA robot (registered via bone fiducials), following previously published methods (18). Intraoperative O-Arm CT scan was used for both registration to bone fiducials and confirmation of final electrode lead position in the CM. RNS-electrodes were automatically pre-localized in native & template space using Lead-DBS software (19) (https://www.lead-dbs.org) and visualized in reference to thalamic nuclei defined by The Thalamus Atlas (20), see Figure 1. The patient recovered and was discharged home on postoperative day 1.

**Figure 2:**
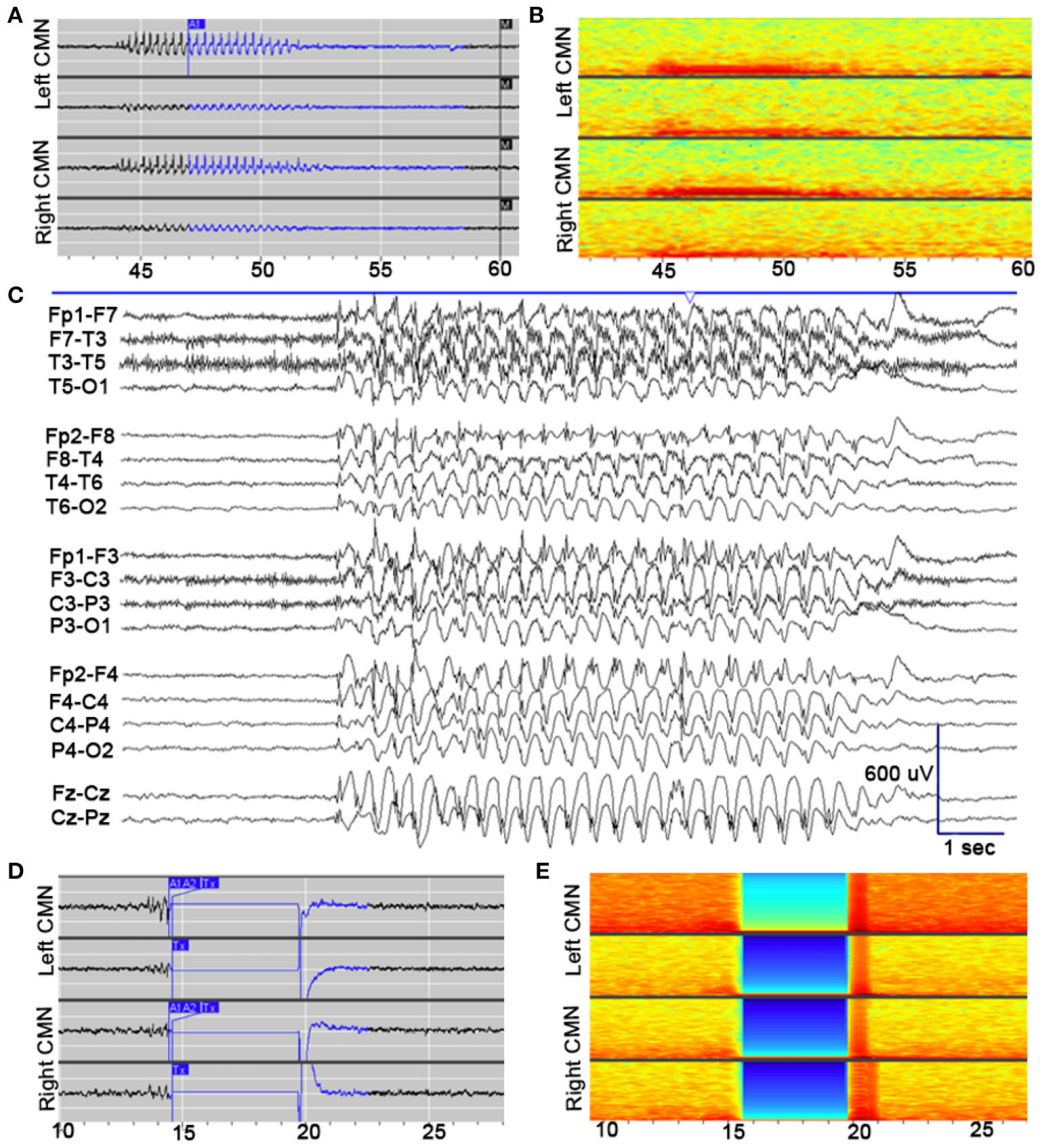
Electrophysiological seizure characteristics. **(A)** Example of an electroclinical seizure stored the NeuroPace Patient Data Management System, detected by device (*A1*) and noted by patient's family with magnet swipe (*M*). Well-formed 3 Hz spike-and-wave discharges are detected maximally in channels 1 (LCM1–LCM2; top row) and 3 (RCM1–RCM2; third row). **(B)** Spectrogram of identical epoch. **(C)** Pre-implantation scalp EEG, capturing electroclinical typical absence seizure (TAS) with behavioral arrest. EEG demonstrates generalized 3 Hz spike- and polyspike-and-wave discharges (longitudinal bipolar montage; sensitivity: 30 μV/mm; timebase 30 mm/s). **(D)** Example of an electroclinical seizure stored the NeuroPace Patient Data Management System, detected by device (*A1, A2*) again detected maximally in channels 1 and 3, with responsive therapy delivered (*Tx*), subsequent amplifier artifact lasting 5 s, and return to electrographic baseline. Therapy delivered to channels 1 and 3: bipolar, current 0.4 mA, frequency 125 Hz, pulse width 160 μs, burst duration 5,000 ms, charge density 0.4 μC/cm^2^. **(E)** Spectrogram of identical epoch.

**Figure 3:**
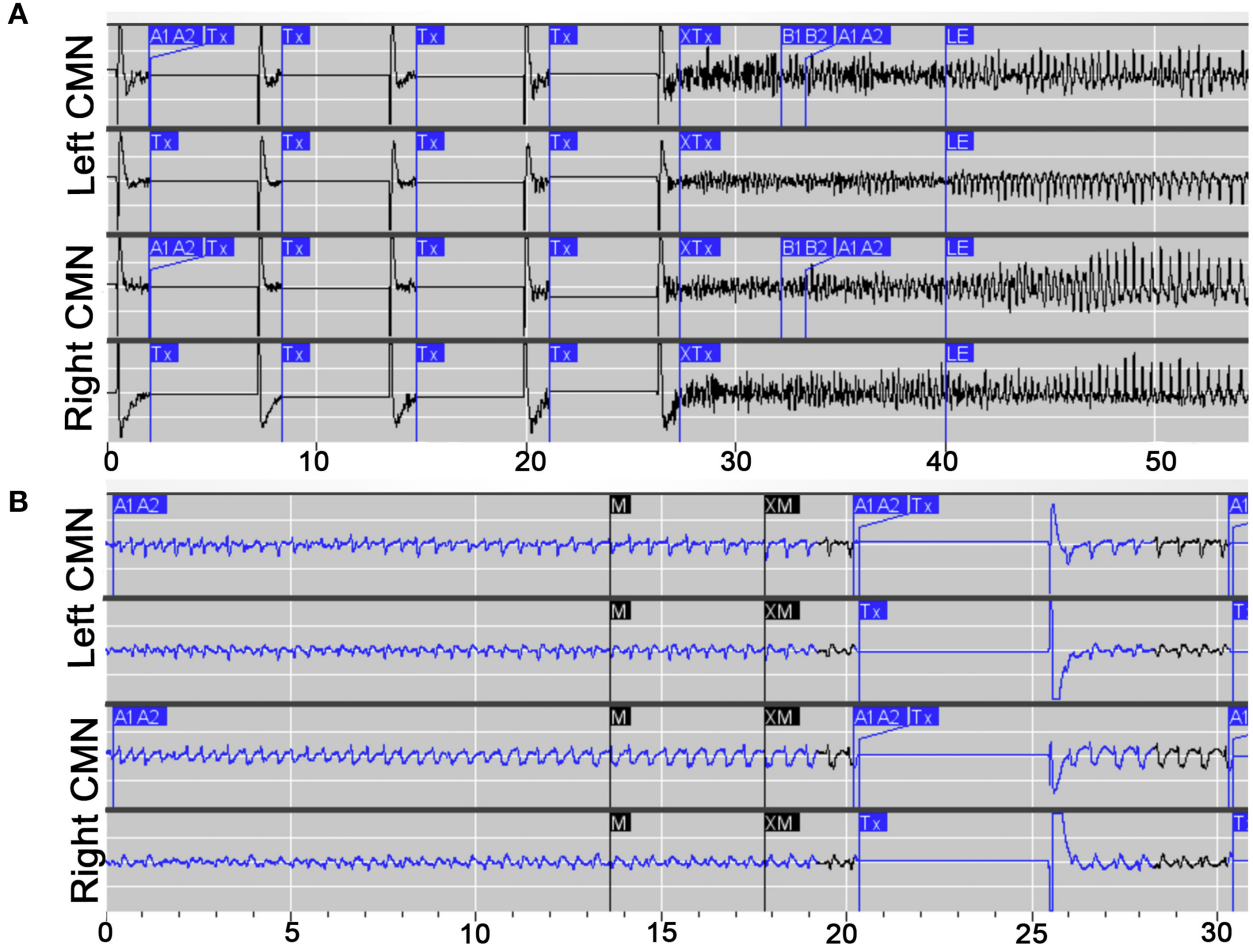
Electrophysiological characteristics of prolonged absence and GTC seizures. **(A)** Example of an electroclinical generalized tonic clonic (GTC) seizure stored the NeuroPace Patient Data Management System, detected by device (A1, A2) and noted by patient's family with magnet swipe (M), with responsive therapy delivered (Tx). **(B)** Example of a prolonged electroclinical absence seizure stored the NeuroPace Patient Data Management System, detected by device (A1, A2) and noted by patient's family with magnet swipe (M), with responsive therapy delivered (Tx).

After implantation, the device was programmed to record scheduled electrocorticography (ECoG), and a broad detector was programmed (75% power change). Multiple ECoG recordings were saved for patient/caregiver event identification (via magnet swipe) over a period of 4 weeks postop. Review of saved ECoG in the Patient Data Management System (PDMS) revealed well-formed 3 Hz spike-wave discharges in the bilateral thalamic contacts, with highest amplitudes in the distal contacts bilaterally (Figure 2). Four weeks after implantation, the detection pattern was adjusted to reliably detect ictal discharges (channel 1: bandpass 2.0–41.7 Hz, amplitude threshold 4%, minimum duration 0.38 s; channel 3: bandpass 2.0–25 Hz, amplitude threshold 5%, minimum duration 0.38 s). Bipolar stimulation of most distal contacts (contact 1 and 2 bilaterally) was enabled at 0.2 μC/Cm^2^ (0.2 mA, 125 Hz, 160 μS for 5,000 ms), in response to detected 3 Hz spike-wave discharges in these channels. Low charge density was used initially due to patient reporting non-painful left arm paresthesia during stimulation. Additionally, ECOG recordings captured prolonged absence seizure and generalized tonic-clonic seizure activity, see Figure 3.

## Follow-Up and Outcomes

One month after turning on stimulation, the patient reported improved seizure frequency, although continued to experience multiple weekly absence seizures. At that time, our patient elected to increase stimulation and was willing to tolerate mild left arm paresthesia along with this this increase to 0.4 μC/Cm^2^ (0.4 mA, 125 Hz, 160 μS for 5,000 ms). These paresthesias resolved within a few days of stimulation. Changes to other stimulation parameters were not considered due to the fact that this patient's symptoms were mild and temporary, but may be considered in other cases (21).

At most recent follow-up 6-months post-implant, the patient and family reported no noticeable absence seizures and reported 1 generalized convulsive seizure per month, improved from previous baseline of 3 to 4 per month. Our patient is tolerating increased stimulation parameters with a charge density of 1.5 μC/Cm^2^ (1.5 mA, 125 Hz, 160 μS for 5,000 ms) without side effects, including resolution of left arm paresthesia. Long Episodes were detected at a rate of 4.4/month, with an average of 441 therapies delivered per day. Repeat scalp EEG has not yet been performed.

## Discussion

Here we describe the first pediatric PGE patient with absence seizures successfully recorded from bilateral CM RNS, and report on successful RNS targeting absence seizures in drug-resistant CAE. This patient experienced decreased seizure frequency at 1-month follow-up, and patient and family reports resolution of detectable absence seizures as well as 75% reduction in generalized convulsive seizures at 6-month follow up. Our findings suggest that CM RNS can prevent loss of consciousness through disruption of low-frequency thalamocortical ictal recruitment.

RNS is a promising technology which offers personalized therapy based on a patient's own seizure electrophysiology by recording and responding to neural activity through delivery of programmable stimulation directly to seizure foci. Several multi-center outcomes studies have demonstrated the efficacy of the RNS system for the treatment of drug-resistant mesial temporal or neocortical seizures, in which 70% of patients saw a 78% reduction in seizure frequency at 6 years (13, 22–24). While the data captured by the RNS system remains computationally intensive to interpret, there are considerable, promising advances being made in the field to improve RNS as a patient-specific therapy (12, 25). In line with this, bilateral centromedian/ventrolateral thalamic RNS in an adult patient was successful in the treatment of generalized epilepsy (eyelid myoclonia with absence) (26). We provide further evidence to support RNS therapy as a safe and effective treatment option for drug-resistant PGE for pediatric patients and the CM as a targetable foci.

The CM receives converging input from the cortex, basal ganglia, and brainstem and participates in cognition (attention and arousal) and sensorimotor coordination (27). Thalamocortical feedback loops regulate cortical input during wakefulness to maintain attention and awareness and its suppression is implicated in the pathogenesis of CAE (27–31). The loss of awareness associated with absence seizures is theorized to occur during electrical perturbations in this feedback loop, such as seen in the aberrant low frequency thalamocortical signaling that is characteristic of absence seizures (29, 30, 32, 33). Neurostimulation of the CM disrupts the low-frequency ictal thalamocortical recruitment and may therefore help to prevent loss of awareness during seizure activity (31, 34). Leveraging the diffuse connectivity profile of this region, the CM has been successfully targeted by DBS for the treatment of drug-resistant PGE (8, 9, 15, 35, 36). RNS stimulation of the CM has been applied in adult patients for the treatment of drug-resistant regional neocortical epilepsy (37), generalized epilepsy (26), Lennox-Gastaut Syndrome (10, 38), and drug-resistant focal onset-seizures (39). Seizure frequency of patients with implanted RNS systems often continues to improve over months to years, which implicates the role of neural plasticity induced by programmable closed-loop stimulation (16, 23). Further research is needed to better understand the mechanisms underlying the clinical benefits of RNS CM stimulation for the treatment of CAE. We show here that the RNS targeting of the CM in this pediatric patient was able to reliably identify ictal discharges and improve seizure frequency through neurostimulation. Other groups have performed RNS of other targets (i.e., anterior thalamic nucleus) for the treatment of generalized epilepsy and the relative efficacy of subcortical RNS targets remains a topic for further investigation (40).

Electrophysiologic studies reveal that clinically relevant ictal discharges can be detected in the CM nucleus (16, 26). Kokkinos et al. (26), performed direct recording of the CM nucleus via RNS showing 3–5 Hz spike and wave activity consistent with their patient's preoperative EEG pattern. Warren et al. (16), performed simultaneous EEG and CM recordings during DBS implantation to examine the relationship between generalized paroxysmal fast activity (GPFA) and slow spike wave (SSW) on EEG and from direct CM recordings. In this study, 86% of GPFA events were seen in both on both scalp EEG and CM, whereas 25% of SSW was observed from both recordings. Interestingly, these recordings suggested that epileptiform activity occurred in cortex prior to CM. Further work will elucidate the interactions of cortex and CM in generalized epilepsy, but these findings suggest that clinically relevant ictal discharges are present in the CM nucleus.

The CM was targeted in this report using indirect and direct technique as previously described (10, 15–17). The CM remains difficult to demarcate on standard neuroimaging (36), however studies have shown that inverse MP2RAGE and quantitative susceptibility mapping (QSM) can be used to identify the nucleus with good reliability (16, 41). Confirmation of electrode placement using intraoperative microelectrode recording (MER) evaluation of the CMN neurophysiological signature has shown mixed results, given the presence of low frequency firing rate while sedated, considerable interpatient variability, and subtle differences in neural signatures between adjacent thalamic nuclei (16). Further research is needed to improve techniques for identifying thalamic subnuclei.

While this case highlights the promising utility of RNS for the treatment of complex, pediatric, drug-resistant PGE with absence seizures, the conclusions drawn are limited by the single patient sample size, short follow-up duration, and the potential under-reporting of absence seizure frequency. Repeat scalp EEG has not been performed in our patient since placement of RNS device, resulting in reliance on patient and family report for clinical absence seizure frequency. Our patient did have other seizure types emerge throughout his course, which precludes classifying his case as pure PGE, and presented unique challenges to his treatment plan. However, he originally presented with and continued to suffer from clearly well-defined typical absence seizures, which are the primary target of his RNS therapy. Successful treatment in our patient's case may highlight the possible role for thalamic RNS therapy in patients with primary generalized epilepsy as well as other cases of complex epilepsy in which cortico-thalamic networks are thought to play a large role.

The development of novel therapies for the treatment of pediatric drug-resistant PGE remain an important area of investigation. Children with PGE experience considerable burden on their quality of life and often experience cognitive, behavioral, and developmental deficits as a result of uncontrolled epilepsy during this critical period of brain development (4–7). Neocortical RNS implantation has been used successfully for the treatment of pediatric drug-resistant seizures in a few cases (42–45). Together, these provide preliminary evidence that RNS is a viable therapeutic option for patients with drug-resistant epilepsy who are not candidates for resective epilepsy surgery.

## Data Availability Statement

The raw data supporting the conclusions of this article will be made available by the authors, without undue reservation.

## Ethics Statement

Written informed consent was obtained from the minor(s)' legal guardian/next of kin for the publication of any potentially identifiable images or data included in this article.

## Author Contributions

All authors listed have made a substantial, direct and intellectual contribution to the work, and approved it for publication.

## Conflict of Interest

TA was a consultant for Monteris Medical and receives research funding through Monteris Medical for the LAANTERN Trial. The remaining authors declare that the research was conducted in the absence of any commercial or financial relationships that could be construed as a potential conflict of interest.
